# Association between polymorphisms in phospholipase A_2_ genes and the plasma triglyceride response to an n-3 PUFA supplementation: a clinical trial

**DOI:** 10.1186/s12944-015-0009-2

**Published:** 2015-02-21

**Authors:** Bénédicte L Tremblay, Hubert Cormier, Iwona Rudkowska, Simone Lemieux, Patrick Couture, Marie-Claude Vohl

**Affiliations:** Institute of Nutrition and Functional Foods (INAF), Laval University, 2440 Hochelaga Blvd, Quebec, QC G1V 0A6 Canada; CHU de Québec Research Center – Endocrinology and Nephrology, 2705 Laurier Blvd, Quebec, QC Canada

**Keywords:** Gene-diet interactions, Plasma lipid levels, Omega-3 fatty acids, Phospholipase, Nutrigenetics

## Abstract

**Background:**

Fish oil-derived long-chain omega-3 (n-3) polyunsaturated fatty acids (PUFAs), including eicosapentaenoic acid (EPA) and docosahexaenoic acid (DHA), reduce plasma triglyceride (TG) levels. Genetic factors such as single-nucleotide polymorphisms (SNPs) found in genes involved in metabolic pathways of n-3 PUFA could be responsible for well-recognized heterogeneity in plasma TG response to n-3 PUFA supplementation. Previous studies have shown that genes in the glycerophospholipid metabolism such as phospholipase A_2_ (PLA_2_) group II, IV, and VI, demonstrate changes in their expression levels in peripheral blood mononuclear cells (PBMCs) after n-3 PUFA supplementation.

**Methods:**

A total of 208 subjects consumed 3 g/day of n-3 PUFA for 6 weeks. Plasma lipids were measured before and after the supplementation period. Five SNPs in *PLA2G2A*, six in *PLA2G2C*, eight in *PLA2G2D*, six in *PLA2G2F*, 22 in *PLA2G4A*, five in *PLA2G6*, and nine in *PLA2G7* were genotyped. The MIXED Procedure for repeated measures adjusted for age, sex, BMI, and energy intake was used in order to test whether the genotype, supplementation or interaction (genotype by supplementation) were associated with plasma TG levels.

**Results:**

The n-3 PUFA supplementation had an independent effect on plasma TG levels. Genotype effects on plasma TG levels were observed for rs2301475 in *PLA2G2C*, rs818571 in *PLA2G2F*, and rs1569480 in *PLA2G4A*. Genotype x supplementation interaction effects on plasma TG levels were observed for rs1805018 in *PLA2G7* as well as for rs10752979, rs10737277, rs7540602, and rs3820185 in *PLA2G4A*.

**Conclusion:**

These results suggest that, SNPs in PLA_2_ genes may influence plasma TG levels during a supplementation with n-3 PUFA. This trial was registered at clinicaltrials.gov as NCT01343342.

## Background

Cardiovascular disease (CVD) is the leading cause of mortality worldwide [1]. Triglyceride (TG) is an independent risk factor of CVD [2]. Fish oil-derived long-chain omega-3 (n-3) polyunsaturated fatty acids (PUFAs), including eicosapentaenoic acid (EPA) and docosahexaenoic acid (DHA) play a significant role in preventing CVD [3]. The potential underlying mechanisms of n-3 PUFAs for reducing CVD risk are related to their hypo-triglyceridemic, anti-inflammatory, anti-atherogenic, and anti-arrhytmic effects [4]. Health organizations around the world currently recommend consumption of EPA and DHA to reduce CVD risk [5-8]. More specifically, The American Heart Association recommends an intake of 2 to 4 g of EPA/DHA per day for patients who need to lower their TG levels [5].

Yet, there is a well-recognized heterogeneity in the plasma TG response to n-3 PUFA supplementation [9]. For example, in the Fish Oil Intervention and Genotype (FINGEN) Study, 31% of all volunteers showed no reduction in plasma TG after taking 1.8 g EPA and DHA per day for 8 weeks [10]. The inter-individual variability observed in the plasma lipid response to an n-3 PUFA supplementation may partly result from genetic variations in genes involved in metabolic pathways of n-3 PUFA [9,11,12]. Understanding the genetic determinants of inter-individual variability to n-3 PUFA supplementation would provide a more rational basis for advising individuals on intake levels likely to achieve optimal reduction in CVD risk [9].

Our team worked on differences in metabolomic and transcriptomic profiles between responders and non-responders to an n-3 PUFA supplementation and found that the lipid metabolism pathways appears to be one of the most different between those two groups [13]. Genes in the glycerophospholipid metabolism such as phospholipase A_2_ (PLA_2_) group II, IV, and VI had changes in their expression levels after n-3 PUFA supplementation [13].

The PLA_2_ represents an important superfamily of enzymes that catalyze the hydrolysis of the ester bond at the sn-2 position of phospholipids to yield non-esterified fatty acids such as AA and lysophospholipids [14]. Usually, these products lead to the generation of a variety of downstream signaling molecules including prostaglandins, leukotrienes, lysophospholipids, platelet activating factor (PAF), and oxidized lipids [15-20]. AA release by PLA_2_ catalytic reaction is the initial and rate-limiting step for the biosynthesis of eicosanoids [21]. Currently these enzymes are classified into six major groups with many subgroups, depending on their functions and cellular locations: the secreted PLA_2_ (sPLA_2_), lipoprotein-associated PLA_2_ (Lp-PLA_2_), cytosolic PLA_2_ calcium-dependent (cPLA_2_), cytosolic PLA_2_ calcium-independent (iPLA_2_), lysosomal PLA_2_ (lPLA_2_) and adipose-specific PLA_2_ (adPLA) [21]. In addition, elevated plasma PLA_2_ activity, likely *sPLA2G2A*, is an independent risk factor for CVD [22,23] and variations on the *PLA2G4A* gene are associated with a CVD phenotype mediated by dietary PUFAs [24].

The objective of the present study is to examine whether genetic variations in PLA_2_ genes influence plasma TG levels of healthy overweight adults following an n-3 PUFA supplementation.

## Methods

### Study population

A total of 254 subjects from the greater Quebec City metropolitan area were recruited to participate in the study between September 2009 and December 2011 via electronic messages sent to university students and employees as well as advertisements in local newspapers. The participants had to be between 18 and 50 years old, have a body mass index (BMI) between 25 and 40 kg/m^2^, be non-smokers, and with no current lipid-lowering medications. They also needed to be free of any thyroid or metabolic disorders requiring treatment, e.g. diabetes, hypertension, severe dyslipidemia, and coronary heart disease (CHD). Subjects were not included if they had taken n-3 PUFA supplements for at least 6 months prior to the beginning of the study. A total of 210 subjects completed the intervention protocol and 208 had plasma TG levels data available for further analyses. The experimental protocol was approved by the Ethics Committees of Laval University Hospital Research Center and Laval University. The trial was registered at clinicaltrials.gov as NCT01343342.

### Study design and diets

First, subjects followed a two-week run-in period during which they received dietary instructions by a trained registered dietitian to achieve the recommendations from Canada’s Food Guide to Healthy Eating. They were asked to apply these dietary recommendations and maintain their body weight stable throughout the protocol. The following instructions were given to ensure stable n-3 PUFA dietary intake: do not exceed two fish or seafood servings per week (maximum 150 g), prefer white flesh fish to fatty fish (examples were given), and avoid enriched n-3 PUFA food such as milk, juices, bread, and eggs. In addition, subjects were not allowed to take n-3 PUFA supplementation (such as flaxseed), vitamins, or natural health products during the protocol. They were also asked to limit their alcohol consumption to two drinks per week.

Second, after the two-week run-in period, subjects received a bottle containing needed n-3 PUFA capsules (Ocean Nutrition, Nova Scotia, Canada) for the following six weeks of supplementation. Subjects had to take five capsules per day (1 g of fish oil concentrate each) providing a total of 3 g of n-3 PUFA (including 1.9 g EPA and 1.1 g DHA) per day. Compliance was assessed from the return of bottles and by measuring the incorporation of EPA and DHA in plasma phospholipids (PL). The participants were asked to report any deviation during the protocol, write down their alcohol and fish consumption as well as the side effects of supplementation. Before each phase, subjects received detailed written and oral instructions on their diet.

A registered dietitian showed the participants how to complete a 3-day (2 weekdays and 1 weekend day) food journal before and after n-3 PUFA supplementation. Nutrition Data System for Research software version 2011 (Nutrition Coordinating Center (NCC), University of Minnesota, Minneapolis, MN, USA) was used to analyse dietary intakes.

### Anthropometric measurements

Body weight, height, and waist girth were measured according to the procedures recommended by the Airlie Conference [25] and were taken before the run-in period as well as before and after n-3 PUFA supplementation. BMI was calculated as weight in kilograms divided by height in meters squared (kg/m^2^).

### Biochemical parameters

Blood samples were collected from an antecubital vein into vacutainer tubes containing EDTA after 12-hour overnight fast and 48-hour alcohol abstinence. Blood samples were drawn before the run-in period to identify and exclude participants with metabolic disorders. Afterwards, the selected participants had blood samples taken before and after the n-3 PUFA supplementation period. Plasma was separated by centrifugation (2,500 g for 10 min at 4°C), and samples were aliquoted and frozen for subsequent analyses. Plasma total cholesterol (TC) and TG concentrations were measured using enzymatic assays [26]. The high-density lipoprotein cholesterol (HDL-C) fraction was obtained after precipitation of very-low density lipoprotein (VLDL) and low-density lipoprotein (LDL) particles in the infranatant with heparin manganese chloride [27]. LDL cholesterol (LDL-C) was calculated with the Friedewald formula [28]. Apolipoprotein B-100 concentrations were measured in plasma by the rocket immune-electrophoretic method of Laurell, as previously described [29]. Plasma C-reactive protein (CRP) was measured by nephelometry (Prospec equipment Behring) using a sensitive assay [30].

### Fatty acid composition of plasma phospholipids

According to a modified Folch method, plasma lipids were extracted with chloroform:methanol (2:1, by volume) [31]. Total PL were separated by thin layer chromatography using a combination of acetic acid and isopropyl ether. Fatty acids of isolated PL were then methylated and capillary gas chromatography was then used to obtain fatty acids profiles. This technique has been previously validated [32].

### SNP Selection and genotyping

SNPs in PLA2G2A, PLA2G2C, PLA2G2D, PLA2G2F, PLA2G4A, PLA2G6, and PLA2G7 were identified with the International HapMap Project SNP database, based on the National Center for Biotechnology Information (NCBI) B36 assembly Data Rel phase II + III, build 126 (Table 1). Tagger procedure in Haploview software V4.2 was used to determine tag SNPs (tSNPs) using a minor allele frequency (MAF) of 5% and pairwise tagging (R^2^ ≥ 0.80). The LD procedure in Haploview V4.2 was then used to examine linkage disequilibrium (LD) between 5 SNPs in PLA2G2A, 6 in PLA2G2C, 8 in PLA2G2D, 6 in PLA2G2F, 22 in PLA2G4A, 5 in PLA2G6, and 9 in PLA2G7 covering all common variations (MAF > 5%) in these genes. Most of the SNPs were in LD (R^2^ ≥ 0.80), and the mean R^2^ was 0.943 for PLA2G2A, 0.974 for PLA2G2C, 1.0 for PLA2G2D, 0.976 for PLA2G2F, 0.975 for PLA2G4A, 0.968 for PLA2G6, and 0.973 for PLA2G7. The SIGMA GenElute Gel Extraction Kit (Sigma-Aldrich Co., St. Louis, MO, USA) has been used to extract genomics DNA. Selected SNPs (Table 1) were genotyped using validated primers and TaqMan probes (Thermo Fisher Scientific, Waltham, MA, USA) [33]. DNA was then mixed with TaqMan Universal PCR Master Mix (Thermo Fisher Scientific.), with a gene-specific primer and probe mixture (predeveloped TaqMan SNP Genotyping Assays; Thermo Fisher Scientific.) in a final volume of 10 μL. Thereafter, genotypes were determined using a 7500 Real-Time PCR System and analyzed using ABI Prism SDS version 2.0.5 (Thermo Fisher Scientific.). Minor allele homozygotes with a genotype frequency <5% were grouped with heterozygotes for statistical analyses.

**Table 1:** **Selected polymorphisms in phospholipase A**_**2**_ **genes**

**Gene**	**dbSNP No.**	**Sequence**	**Position**	**Allele frequency**	
*PLA2G2A*	rs876018	ATAC[A/T]TGAT	3-UTR	A (n = 352) 0.8421	T (n = 66) 0.1579
	rs955587	GCGT[A/G]GACT	Intron	G (n = 352) 0.8381	A (n = 68) 0.1619
	rs3753827	GTAA[G/T]GCCC	Intron	G (n = 233) 0.5574	T (n = 185) 0.4426
	rs11573156	GGAG[C/G]AGCT	5-UTR	C (n = 326) 0.7762	G (n = 94) 0.2238
	rs11573142	ATGG[C/T]ATTC	NearGene-5	T (n = 406) 0.9667	C (n = 14) 0.0333
*PLA2G2C*	rs6426616	AGCC[A/G]GCCC	Missense Q [Gln]- > R [Arg]	G (n = 249) 0.5929	A (n = 171) 0.4071
	rs12139100	GGGG[C/T]GAAG	Stop-gain	C (n = 356) 0.8476	T (n = 64) 0.1524
	rs10916716	ACCC[A/G]GGCC	Intron	A (n = 358) 0.8524	G (n = 62) 0.1476
	rs2301475	GGAG[A/G]TATT	Intron	A (n = 294) 0.7000	G (n = 98) 0.3000
	rs10916712	GAAG[G/C]TGTG	3-UTR	C (n = 322) 0.76677	G (n = 98) 0.2333
	rs10916718	GCTC[A/G]AAGC	Intron	A (n = 237) 0.5643	G (n = 183) 0.4357
*PLA2G2D*	rs578459	TATC[A/T]TCCA	3-UTR	A (n = 234) 0.5571	T (n = 186) 0.4429
	rs16823482	ATTT[T/C]TCAC	Intron	T (n = 399) 0.9500	C (n = 21) 0.0500
	rs3736979	ACTG[G/A]GTGC	Intron	G (n = 309) 0.7357	A (n = 111) 0.2643
	rs584367	GTGC[A/G]GCAT	Missense S [Ser] - > G [Gly]	G (n = 262) 0.6238	A (n = 158) 0.3762
	rs12045689	GGAG[T/C]AAGA	Intron	T (n = 381) 0.9071	C (n = 39) 0.0929
	rs679667	CCCC[G/A]CTGC	Intron	G (n = 381) 0.9071	A (n = 39) 0.0929
	rs17354769	AACT[A/G]GGGC	NearGene-5	A (n = 399) 0.9545	G (n = 19) 0.0455
	rs10916711	CTAG[T/C]GATT	Intron	T (n = 266) 0.6425	C (n = 148) 0.3575
*PLA2G2F*	rs12065685	GGGC[C/T]TCTG	Non-coding exon	T (n = 369) 0.8786	C (n = 51) 0.1214
	rs6657574	TGAC[C/T]TTGC	Non-coding exon	C (n = 345) 0.8214	T (n = 75) 0.1786
	rs11582551	ATCT[C/T]CTGT	Intron	T (n = 303) 0.7214	C (n = 117) 0.2786
	rs818571	CGCC[C/T]GGAC	3-UTR	C (n = 296) 0.7048	T (n = 124) 0.2952
	rs631134	ATTC[G/A]GTGA	NearGene-5	G (n = 335) 0.7976	A (n = 85) 0.2024
	rs11583904	TGAG[A/G]TGGA	Intron	A (n = 71) 0.169	G (n = 349) 0.831
*PLA2G4A*	rs979924	TACA[C/T]TGCA	NearGene-5	C (n = 33) 0.0786	T (n = 387) 0.9214
	rs2076075	ATTC[G/A]TATAC	Intron	G (n = 381) 0.9071	A (n = 39) 0.0929
	rs3736741	TTCC[A/G]GGCT	Intron	A (n = 320) 0.7619	G (n = 100) 0.2381
	rs10911949	CTAA[C/T]GGCA	Intron	C (n = 222) 0.5286	T (n = 198) 0.4714
	rs10752979	TCTC[A/G]TTGT	Intron	A (n = 68) 0.1619	G (n = 352) 0.8381
	rs1160719	TTTC[A/G]TTAT	Intron	A (n = 79) 0.1881	G (n = 341) 0.8119
	rs10737277	ATCA[C/G]ACAC	Intron	C (n = 231) 0.55	G (n = 189) 0.45
	rs12720702	AATA[A/G]CAAG	Intron	A (n = 386) 0.919	G (n = 34) 0.081
	rs7522213	ATTA[C/T]ATCC	Intron	C (n = 403) 0.9595	T (n = 17) 0.0405
	rs7540602	CTCT[G/T]GACA	Intron	G (n = 379) 0.9024	T (n = 41) 0.0976
	rs10157410	TTTT[C/G]ACTA	Intron	C (n = 57) 0.1357	G (n = 363) 0.8643
	rs12720497	CCAG[C/T]GACC	Intron	C (n = 262) 0.6238	T (n = 158) 0.3762
	rs4651331	CAAG[G/T]AGCA	Intron	G (n = 101) 0.2405	T (n = 319) 0.7595
	rs1569480	TCAC[A/G]ATGG	Intron	A (n = 236) 0.5619	G (n = 184) 0.4381
	rs10911935	ACTC[G/T]TGAT	Intron	G (n = 337) 0.8024	T (n = 83) 0.1976
	rs12353944	AAAA[C/T]CTGA	Intron	C (n = 76) 0.181	T (n = 344) 0.819
	rs11576330	CACA[C/T]CCAC	Intron	C (n = 38) 0.0905	T (n = 382) 0.9095
	rs10489410	TTTC[G/T]TAGT	Intron	G (n = 16) 0.0381	T (n = 404) 0.9619
	rs10911946	TTAG[C/T]TGAC	Intron	C (n = 299) 0.7119	T (n = 121) 0.2881
	rs3820185	CATG[G/T]TGAG	Intron	G (n = 283) 0.3262	T (n = 137) 0.3262
	rs12746200	CCAG[A/G]ATCA	Intron	A (n = 384) 0.9143	G (n = 36) 0.0857
	rs11587539	TAGG[A/T]TTTG	Intron	A (n = 243) 0.5786	T (n = 177) 0.4214
*PLA2G6*	rs5750546	TAAA[G/A]GAAA	Intron	G (n = 259) 0.6167	A (n = 161) 0.3833
	rs132989	GGGG[G/A]ACAG	Intron	G (n = 392) 0.9333	A (n = 28) 0.0677
	rs133016	AGTG[G/A]ACCC	Intron	G (n = 215) 0.5119	A (n = 205) 0.4881
	rs2235346	TGCC[C/A]GGGG	Intron	C (n = 200) 0.4762	A (n = 220) 0.5238
	rs2284060	AATC[A/G]ACGC	Intron	A (n = 228) 0.5429	G (n = 192) 0.4571
*PLA2G7*	rs12195701	ATGT[G/A]GATC	Intron	G (n = 333) 0.7929	A (n = 87) 0.2071
	rs12528807	CCAC[A/C]GATC	Intron	A (n = 379) 0.9024	C (n = 41) 0.0976
	rs1421368	ATGA[C/T]CTTA	Intron	C (n = 34) 0.081	T (386) 0.919
	rs1421378	TGAT[T/C]CGGA	NearGene-5	T (n = 244) 0.581	C (n = 176) 0.419
	rs17288905	TCCA[T/C]AGCA	Intron	T (n = 378) 0.9214	C (n = 33) 0.0786
	rs1805017	GATC[G/A]CCTT	Missense R [Arg] - > H [His]	G (n = 304) 0.7238	A (n = 116) 0.2762
	rs1805018	GAAA[T/C]AGGG	Missense I [Ile] - > T [Thr]	T (n = 403) 0.9595	C (n = 17) 0.0405
	rs6929105	TGAA[A/G]GATG	Intron	A (n = 98) 0.2333	G (n = 322) 0.7667
	rs7756935	GGGG[G/T]TAGA	Intron	G (n = 85) 0.2024	T (n = 335) 0.7976

Allelic frequencies were obtained using the ALLELE Procedure (SAS Genetics v9.3).

### Statistical analyses

All statistical analyses were performed with SAS Statistical Software V9.3 (SAS Institute, Cary, N.C., USA), except for the ALLELE Procedure, which was done with SAS Genetics V9.3. The ALLELE Procedure was used to verify departure from the Hardy-Weinberg equilibrium (HWE) and calculate MAF. Values that were not normally distributed were log_10_ or negative reciprocal transformed before analysis. ANOVA was used to test for significant differences in metabolic characteristics between men and women at baseline with age, sex, and BMI included in the model. A paired t-test was used to test for significant differences between various nutrient intakes before and after n-3 PUFA supplementation. A linear regression using the stepwise bidirectional elimination approach was applied to assess which SNPs could explain part of the plasma TG level variance. The 61 SNPs were in HWE. First, the MIXED procedure for repeated measures was used to test for the effects of the genotype, supplementation and genotype × supplementation interaction on plasma TG in a model adjusted for age, sex, BMI, and energy intake. Secondly, ANOVAs adjusted for age, sex, BMI, energy intake, and pre-supplementation plasma TG levels were used to test the differences in TG levels after supplementation between genotypic groups. Statistical significance was defined as *p* ≤ 0.05.

## Results

Allele frequencies of selected SNPs are shown in Table 1. All SNPs were in HWE. Therefore, associations with 61 SNPs were tested in statistical analyses. The percent coverage was 90% for *PLA2G2A*, 85% for *PLA2G2C*, 90% for *PLA2G2D*, 80% for *PLA2G2F*, 85% for *PLA2G4A*, 98% for *PLA2G6*, and 93% for *PLA2G7*. While most of the SNPs selected were intronic, one *PLA2G2C* SNP, one *PLA2G2D* SNP, and two *PLA2G7* SNPs were located in exons and resulted in amino acid changes: rs6426616 (Gln → Arg), rs584367 (Ser → Gly), rs1805017 (Arg → His), and rs1805018 (Ile → Thr).

Baseline characteristics of the study participants are presented in Table 2. As required by inclusion criteria, men and women were overweight (mean BMI > kg/m^2^) and had mean plasma TG levels slightly above the cut-off value of 1.13 mmol/l recommended by the American Heart Association (AHA) for optimal plasma TG levels [34]. Significant gender differences were observed for weight, HDL-C, TG, and CRP levels. Daily energy and nutrient intakes measured by a 3-day food record are presented in Table 3. After n-3 supplementation, energy, carbohydrate, protein, and saturated fat intakes including n-3 supplements were significantly different from the pre-supplementation period (p = 0.006, p < 0.0001, p = 0.002, and p < 0.0001 respectively). PUFA intakes after the supplementation (including fish oil capsules and food) were significantly higher (p = 0.0002).

**Table 2:** **Baseline characteristics of the study sample (n = 208)**

**Characteristics**	**All**	**Men**	**Women**	***p*** ^***a***^
Study population, n	208	96	112	
Age, years	30.8 ± 8.7	31.2 ± 8.1	30.5 ± 9.1	0.55
Weight, kg^b,d^	81.4 ± 13.9	87.2 ± 13.4	76.4 ± 12.3	<0.0001*
BMI^b,d^	27.8 ± 3.7	27.5 ± 3.6	28.2 ± 3.8	0.13
Waist circumference, cm	93.3 ± 10.8	94.8 ± 11.0	92.0 ± 10.4	0.06
TC, mmol/L^e^	4.82 ± 1.01	4.80 ± 1.00	4.83 ± 1.02	0.75
HDL-C, mmol/L^e^	1.46 ± 0.39	1.29 ± 0.31	1.61 ± 0.39	<0.0001*
LDL-C, mmol/L^e^	2.79 ± 0.87	2.91 ± 0.87	2.70 ± 0.86	0.08
TG, mmol/L^b,e^	1.23 ± 0.64	1.32 ± 0.74	1.15 ± 0.53	0.04*
Apolipoprotein B, g/l^e^	0.86 ± 0.25	0.89 ± 0.25	0.84 ± 0.25	0.12
CRP, mg/L^c,e^	3.13 ± 7.10	1.66 ± 2.45	4.39 ± 9.24	0.02*

Values are means ± SD. *p < 0.05.

^a^p value from ANOVA for the differences between men and women at baseline;

^b^values are log_10_ transformed;

^c^values are negative reciprocal transformed;

^d^values adjusted for age;

^e^values adjusted for age and BMI.

**Table 3:** **Nutrient intakes before and after n-3 PUFA supplementation (n = 208)**

**Dietary Intakes**	**Pre-supplementation**	**Post-supplementation**	***P-*** **values** ^*^
		**(including** ***n*** **-3 PUFA supplements)**	
Energy (kcal)	2273 ± 590	2186 ± 566	0.006
Carbohydrate (g/d)	286.7 ± 78.9	263.4 ± 77.7	<0.0001
Protein (g/d)	97.8 ± 30.2	92.6 ± 29.6	0.002
Total fat (g/d)	84.5 ± 29.2	86.6 ± 29.8	0.44
SFA (g/d)	29.0 ± 12.0	25.4 ± 10.4	<0.0001
MUFA (g/d)	30.8 ± 11.8	29.6 ± 12.4	0.11
PUFA (g/d)	15.2 ± 6.6	17.1 ± 6.9	0.0002

Values are means ± SD. p < 0.05. *p-values provided by a paired t-test.

MUFA = monounsaturated fatty acids; PUFA = polyunsaturated fatty acids; SFA = saturated fatty acids.

Subjects were asked to limit their fish intake to no more than two servings per week (one serving of fish = 75 g). Based on the compliance questionnaire, the mean intake of fish was of 0.89 serving per week during the n-3 PUFA supplementation period. Accordingly, subjects who had consumed the maximum quantity of fish permitted each week (150 g) would have had an extra 0.43 g of EPA and DHA per day. Following the supplementation, TG levels decreased in 71.2% and increased in 28.8% of the subjects (delta mean ± SD = −0.25 ± 0.15 and 0.20 ± 0.19 mmol/l, respectively), as previously reported [35].

We further tested the independent effect of the genotype, the supplementation or the interaction (genotype by supplementation) on plasma TG levels. First, the supplementation had an independent effect on plasma TG levels (p < 0.0001), as expected. Secondly, three SNPS, one of *PLA2G2C* (rs2301475), one of *PLA2G2F* (rs818571), and one of *PLA2G4A* (rs1569480) were associated with plasma TG levels. Thirdly, interaction effects between n-3 PUFA supplementation and genotype were observed for one SNP of *PLA2G7* (rs1805018) and four of *PLA2G4A* (rs10752979, rs10737277, rs7540602, and rs3820185) (Table 4). All associations remained significant after further adjustments for changes in carbohydrate, protein and saturated fat intakes and for changes in PUFA levels in plasma phospholipids (data not shown).

**Table 4:** **Significant effects of the genotype, the n-3 supplementation and the genotype x supplementation on TG levels (n = 208)**

**Genes**	**SNPs**	**Genotype**	**Supplementation**	**Interaction**
		***p***	***p***	***p***
***PLA2G2C***	rs2301475	0.0209	<.0001	0.8703
***PLA2G2F***	rs818571	0.0188	<.0001	0.3958
***PLA2G7***	rs1805018	0.2383	<.0001	0.0286
***PLA2G4A***	rs10752979f	0.9152	<.0001	0.0273
***PLA2G4A***	rs10737277	0.6696	<.0001	0.0241
***PLA2G4A***	rs7540602f	0.1663	<.0001	0.0344
***PLA2G4A***	rs1569480	0.0203	<.0001	0.7758
***PLA2G4A***	rs3820185	0.4778	<.0001	0.0231

*p values are derived from log_10_-transformed data.

All results were adjusted for age, sex, BMI, and energy intake.

The MIXED procedure (SAS v9.3) was used to test the interaction effects.

**Figure 1:**
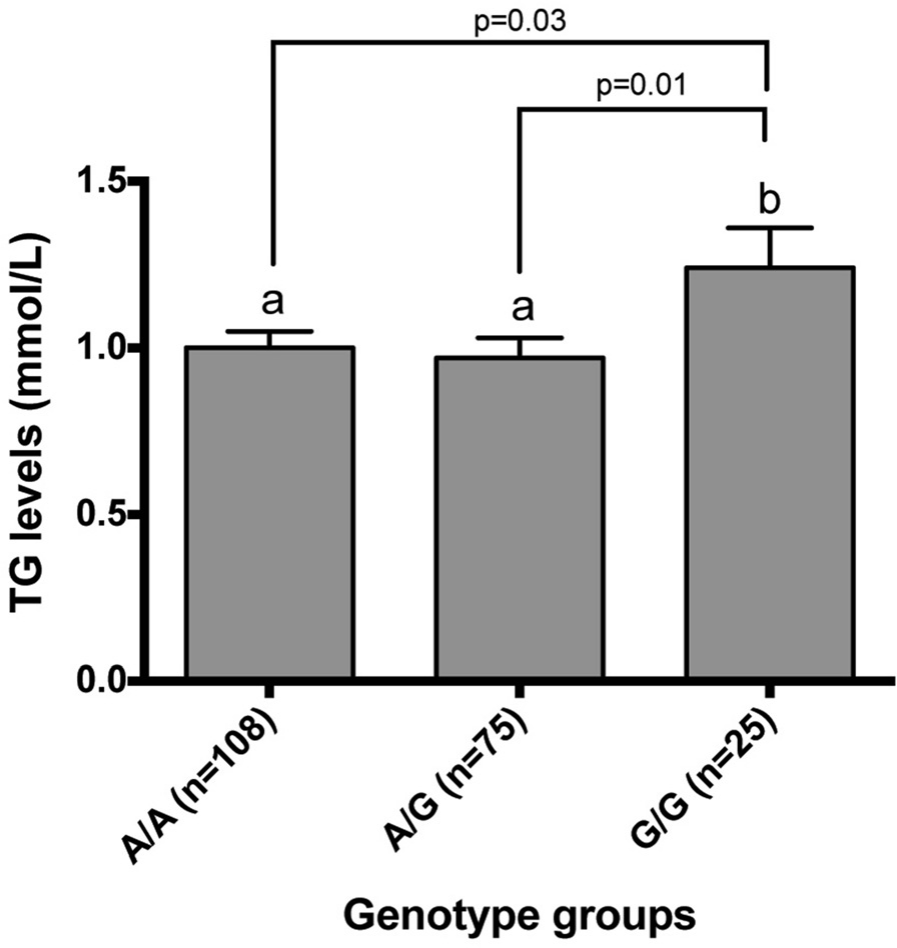
**TG levels after an n-3 PUFA supplementation by genotype groups for rs2301475 (** ***PLA2G2C*** **).**

**Figure 2:**
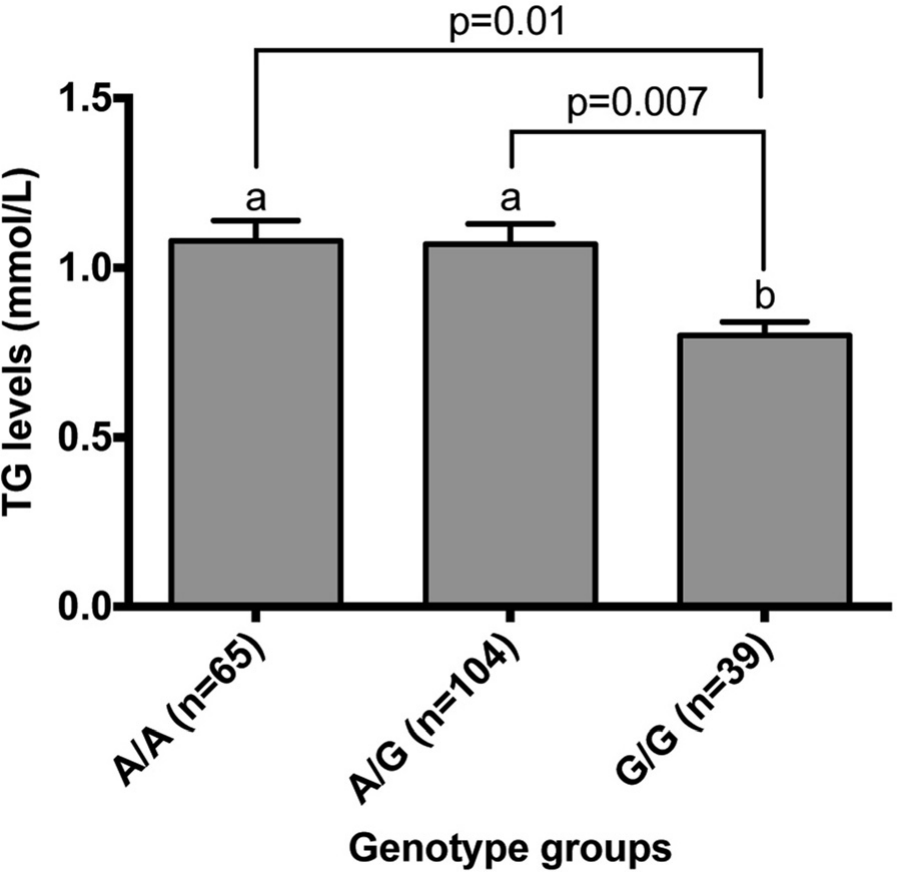
**TG levels after an n-3 PUFA supplementation by genotype groups for rs1569480 (** ***PLA2G4A*** **).**

Further analyses revealed that post-supplementation plasma TG levels were different only for rs2301475 (*PLA2G2C*) and rs1569480 (*PLA2G4A*) but not for rs818571 (PLA2G2F). Figures 1 and 2 show significant differences between post-supplementation plasma TG levels of genotype groups for rs2301475 and rs1569480 after adjustments for age, sex, BMI, and energy. However, the association was no longer significant for these two SNPs after adjustment for pre-supplementation plasma TG levels.

Finally, 60 SNPs were included in a linear regression model, post-supplementation plasma TG levels as the dependant variable, adjusted for pre-supplementation plasma TG levels, age, sex, BMI, and energy intake. Using the stepwise bidirectional selection method, SNPs in *PLA2G2D*, *PLA2G7*, and *PLA2G4A* were associated (p ≤ 0.05) with post-supplementation TG levels. Rs132989 from *PLA2G6*, rs1805018 from *PLA2G7*, rs679667 and rs12045689 from *PLA2G2D* and rs10752979 and rs1160719 from *PLA2G4A* explained, respectively 1.06, 1.14, 0.98, 0.63, 1.27, and 0.82% of the trait. In sum, SNPs on those genes explain 5.9% of the trait.

## Discussion

In this study, we tested whether the plasma TG levels response of healthy overweight adults to n-3 PUFA supplementation is modulated by genes encoding PLA_2_. Our team observed that *PLA2G2A*, *PLA2G2C*, *PLA2G2D*, *PLA2G2F*, *PLA2G4A,* and *PLA2G6* are modulated by n-3 PUFA supplementation, since they were differentially expressed in peripheral blood mononuclear cells (PBMCs) after supplementation [13]. PLA_2_ family was shown to be influenced by n-3 PUFA supplementation so we included *PLA2G7* since its gene product is a secreted enzyme whose activity is associated with CHD biomarkers [36,37].

In the present study, we tested the independent effects of the supplementation, genotypes of selected SNPs in PLA_2_ genes, and genotype x supplementation interaction on plasma TG levels. As expected, n-3 PUFA supplementation significantly lowered plasma TG levels, a finding that is concordant with results reported in literature [10,38]. Moreover, three SNPs of PLA_2_ genes influenced TG levels independently of the supplementation. In addition, genotypes x supplementation interaction effects were observed for five SNPs as previously mentioned. These SNPs and interaction effects considerably contributed to explain inter-individual variability in plasma TG levels after n-3 PUFA supplementation. Despite the fact that some nutrient intakes were significantly different pre- and post-supplementation, further analyses taking into account changes in carbohydrate, protein and saturated fat intakes revealed that results remained the same (data not shown). SNPs of *PLA2G2D, PLA2G7,* and *PLA2G4A* explained 5.9% of the variance in post TG supplementation, in a linear regression model.

Our study showed that genetic factors, especially SNPs of *PLA2G2C*, *PLA2G2F*, *PLA2G4A,* and *PLA2G7* influenced plasma TG levels response to n-3 supplementation and therefore potentially explained the variability observed which is consistent with findings from other investigators [9,10,38]. *PLA2G2C* and *PLA2G2F* that are part of the sPLA_2_ group have been less studied. *PLA2G2C* appears to be a non-functional pseudogene, unlike its rodent counterpart [39]. *PLA2G2F* has a twofold preference for AA over linoleic acid *in vitro* and that its expression generally increases in the aorta consecutively with advance of atherosclerosis [40,41].

*PLA2G7* encodes human Lp-PLA_2_, also known as platelet-activating factor acetylhydrolase (PAF-AH), which has been much largely studied in the literature [21]. Lp-PLA_2_ may play an important role in the pathophysiology of inflammation because it participates in the oxidative modification of LDL [42]. High levels of Lp-PLA_2_ mass and activity were associated with the risk of CHD, stroke, and cardiovascular mortality [43]. It also may be an emerging biomarker for improved cardiovascular risk assessment in clinical practice and a potential therapeutic target for primary and secondary prevention of CVD [43]. The rs1805017 G and rs1051931 A alleles of *PLA2G7* gene were found to be associated with coronary artery disease (CAD) [44] yet, up to now, studies are inconclusive on the association between *PLA2G7* variants and cardiovascular risk [43,45-47]. SNP rs1805018 tended toward significantly decreased expression of the *PLA2G7* gene [44] and decreased Lp-PLA_2_ activity [48].

One of our selected SNPs, rs1805018 (I198T), was part of the SNPs that had a genotype x supplementation interaction and was in strong-LD with SNPs found in the literature, namely rs201554087 (V279P), rs1051931 (A379V), and rs1805017 (R92H) [48]. Consequently, we may suppose that the genotype x supplementation effect we observed with rs1805018 is partly the reflection of these other functional SNPs. Further analyses performed with ESEfinder 3.0 showed that rs1805018 located in the coding region may impact mRNA splicing. However, analyses with SIFT and PolyPhen-2 did not confirm the potential functional effect of this SNP since the amino acid change was considered tolerated or benign.

Therefore, the majority of genotype x supplementation effects were observed with SNPs within *PLA2G4A* gene (rs10752979, rs10737277, rs7540602, and rs3820185). *PLA2G4A* encodes a cPLA_2_ that is now considered a central enzyme for mediating eicosanoid production and thus plays a major role in inflammatory diseases. Indeed, *PLA2G4A* hydrolysis of phospholipid substrates has high substrate specificity for AA at the sn-2 position [21]. The release of AA has been linked to the action of cPLA_2_ but release of DHA is less clear, although the action of iPLA_2_ has been suggested in literature [49,50]. In addition, a functional variant (rs12746200) was associated with CVD phenotype mediated by dietary PUFAs [24]. Interestingly, our team demonstrated that participants who did not lower their TG levels (non-responders) had lower *PLA2G4A* expression after n-3 PUFA supplementation and that PLA2G4A was expressed in opposite direction between responders and non-responders after supplementation [13]. We could postulate that a lower *PLA2G4A* expression may decrease the release of EPA, DHA, and AA from cellular membrane and thus decrease activation of peroxisome proliferator-activated receptors alpha (PPAR-α) and PPAR-γ and their action to reduce TG levels [51-53]. Indeed, n-3 and AA activate PPAR-α to decrease TG and VLDL secretion and increase fatty acid oxidation in the liver. N-3 and AA also activate PPAR-γ in the adipose tissues to improve insulin sensitivity, increasing TG clearance, supressing lipolysis and hepatic TG production, thus helping to decrease TG levels [54-56].

## Conclusions

Data from the present study suggest that SNPs within PLA_2_ genes may modulate plasma TG levels after n-3 PUFA supplementation in healthy overweight adults. These results need to be replicated in other independent studies, therefore we will be able to better understand the potential functional mechanism underlying these genetic associations. In conclusion, these results indicate that gene-diet interaction effects may modulate the response of plasma TG levels to n-3 PUFA intakes and thus contribute to the explanation of the inter-individual variability observed.

## Consent

Written informed consent was obtained from all subjects for the publication of this report.
